# Genetic Parameters of Semen Traits and Their Correlations with Conformation Traits in Chinese Holstein Bulls

**DOI:** 10.1155/2024/5593703

**Published:** 2024-01-29

**Authors:** Xiao Wang, Jian Yang, Jie Xue, Miao Zhang, Fan Zhang, Kun Wang, Yanqin Li, Yuanpei Zhang, Xiaoping Wu, Feng Wang, Xiuxin Zhao, Junqing Ni, Yabin Ma, Rongling Li, Lingling Wang, Guosheng Su, Yundong Gao, Jianbin Li

**Affiliations:** ^1^Institute of Animal Science and Veterinary Medicine, Shandong Academy of Agricultural Sciences, Jinan 250100, China; ^2^College of Animal Science and Veterinary Medicine, Shandong Agricultural University, Tai'an 271018, China; ^3^Shandong OX Livestock Breeding Co., Ltd., Jinan 250100, China; ^4^Fine Breed Centre of Animal Husbandry of HeBei, Shijiazhuang 050061, China

## Abstract

The elite bull plays an extremely important role in the genetic progression of the dairy cow population. The previous results indicated the potential positive relationship of large scrotal circumference (SC) with improved semen volume, concentration, and motility. In order to improve bull's semen quantity and quality by selection, it is necessary to estimate the genetic parameters of semen traits and their correlations with other conformation traits such as SC that could be used for an indirect selection. In this study, the genetic parameters of seven semen traits (*n* = 66,260) and nine conformation traits (*n* = 3,642) of Holstein bulls (*n* = 453) were estimated by using the bivariate repeatability animal model with the average information-restricted maximum likelihood (AI-REML) approach. The results showed that the estimated heritabilities of semen traits ranged from 0.06 (total number of motile sperm, TNMS) to 0.37 (percentage of abnormal sperm, PAS) and conformation traits ranged from 0.23 (pin width, PW) to 0.69 (hip height, HH). The highest genetic correlations were found between semen volume per ejaculation (SVPE), semen concentration per ejaculation (SCPE), total number of sperm (TNS), and TNMS traits that were 0.97, 0.98, 1.00, and 0.99, respectively. Phenotypic correlations between SC and SVPE, SCPE, TNS, and TNMS were 0.35, 0.35, 0.48, and 0.42, respectively. In summary, the moderate or high heritability of semen traits indicates that genetic improvement of semen quality by selection is feasible, where SC could be a useful trait for indirect selection or as correlated information to improve semen quantity and production in the practical bull breeding programs.

## 1. Introduction

The widespread use of frozen semen from bulls for artificial insemination contributes 70% to the genetic progression in dairy cattle [1]. As semen qualities directly determine the conception rates, the reproductive performances of bulls require to be continually improved to supply high-quality semen for efficient production. Therefore, selection is necessary for high-quality and large-quantity semen from genetically superior sires [2, 3]. Meyer et al. [4] estimated the genetic correlations between reproduction and growth traits in Australian beef cattle and suggested the indirect selection of larger scrotal circumference (SC) sizes for the shorter calving intervals [4]. In addition, other conformation traits such as body weight have positive correlations with viable sperm cell production that can also be used for the selection of reproduction traits [5]. Bulls' breeding fitness may perform superiorly if their morphological conformation is better as well, so bull conformation traits have potential influences on semen production and quality [6]. Thus, understanding the heritability of these traits is crucial for effective selection programs.

In the previous studies, Nickolov and Metodiev [7] found the heritabilities of semen volume, motility, and concentration traits equal to 0.43, 0.54, and 0.61, respectively, in Holstein bulls. However, Druet et al. [8] presented a relatively low heritability of semen volume (0.15) but a moderate heritability of concentration (0.30) and a relatively high heritability of motility (0.59). Of note, a significant positive effect of SC on semen volume, concentration, and motility can be identified, for example, the significant genetic correlations with volume (0.20), concentration (0.77), and motility (0.34) in Hereford bulls [9]. In young Nellore bulls, a low negative genetic correlation (−0.24) of SC with the percentage of total sperm defects was observed [10].

The inconsistent genetic parameters of these previous studies could be caused by heterogeneous datasets, different genetic and environmental backgrounds, and various statistical models among different populations [11]. It becomes apparent that further estimations of genetic parameters in one population are needed to guarantee accurate breeding programs in the dairy industry. Until now, correlation studies between semen traits and other conformation traits such as body length and chest girth are also scarce [12]. Therefore, our study aims to estimate the genetic parameters of semen traits and body conformation traits in Chinese Holstein bulls to investigate the possible selection methods to improve semen quality. The obtained estimation results could be used for genetic improvement of semen production and quality in practical selection programs of the Holstein population.

## 2. Materials and Methods

### 2.1. Semen and Conformation Traits

**Figure 1:**
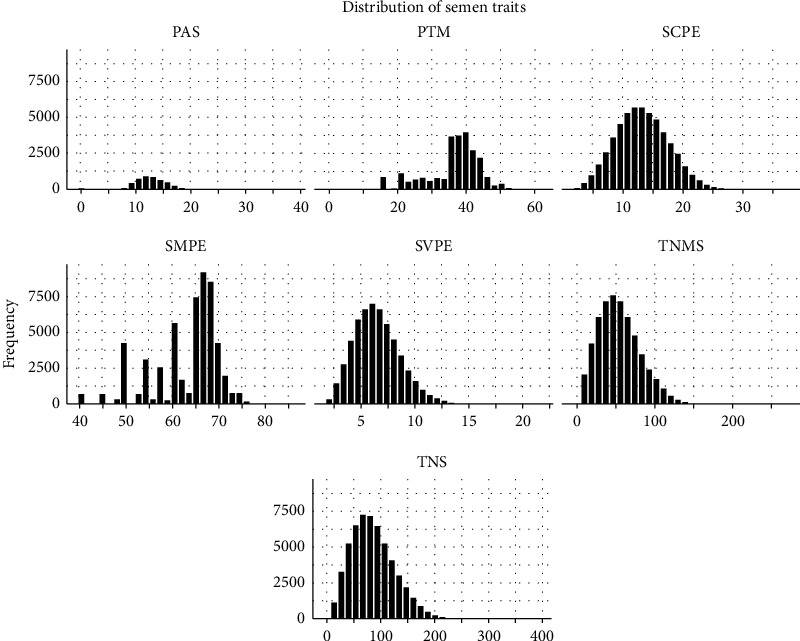
The distribution of seven semen traits. SVPE = semen volume per ejaculation, SMPE = sperm motility per ejaculation, SCPE = semen concentration per ejaculation, PAS = percentage of abnormal sperms, TNS = total number of sperms, TNMS = total number of motile sperms, and PTM = postthaw motility.

**Figure 2:**
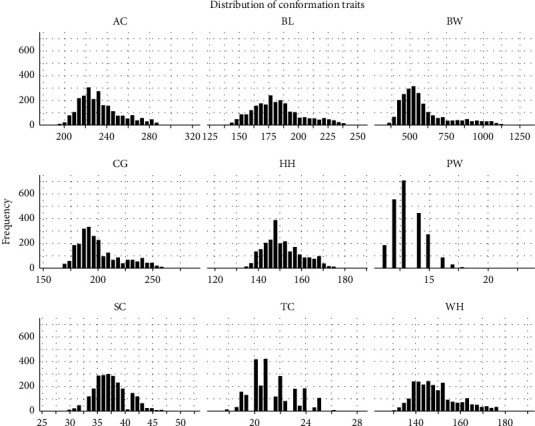
The distribution of nine conformation traits. BW = body weight, WH = withers height, BL = body length, CG = chest girth, AC = abdominal circumference, TC = tube circumference, PW = pin width, HH = hip height, and SC = scrotal circumference.

The phenotype records of semen (*n* = 66,260) and conformation (*n* = 3,642) traits were collected from 453 Chinese Holstein bulls over the last 10 years from Shandong OX Livestock Breeding Co., Ltd., in Shandong province, China. After several quality control procedures, 56,980 semen records and 2,635 conformation records were retained for further analysis (Table 1). The bulls were raised in their own management systems and fed with customized pellet feed and high-quality green hay (*Leymus chinensis*, alfalfa, and oats) with the mineral supplement. All bulls were vaccinated and treated based on the guidelines and recommendations of the veterinarian. The distributions of seven semen traits and nine conformation traits are shown in Figures 1 and 2, respectively. The detailed information on them is listed in Supplementary File 1.

The seven semen traits included semen volume per ejaculation (SVPE), semen concentration per ejaculation (SCPE), sperm motility per ejaculation (SMPE), total number of sperm (TNS), total number of motile sperm (TNMS), percentage of abnormal sperm (PAS), and postthaw motility (PTM). Semen was collected twice a week from each bull by using an artificial vagina and then immediately stored at 37°C in a water bath. The fresh semen traits of SVPE (mL), SCPE (10^8^/mL), SMPE (%), and PAS (%) were evaluated by the skilled technicians, where TNS = SVPE (mL) × SCPE (10^8^/mL) and TNMS = TNS (10^8^) × SMPE (%). After 5–7 days of cryopreservation, two straws of each bull were randomly taken and thawed at 38°C for 20 seconds. Immediately, PTM (%) was evaluated under a light microscope.

The nine conformation traits were generally measured in the middle of the month which included body weight (BW), withers height (WH), body length (BL), chest girth (CG), abdominal circumference (AC), tube circumference (TC), pin width (PW), hip height (HH), and scrotal circumference (SC). WH (cm) was measured from the highest point of the armor to the ground as the vertical distance. BL (cm) was measured from the shoulder to the end of the sciatic bone. CG (cm) was measured as the vertical circumference of the body at the posterior scapula. TC (cm) was measured as the upper third of the tube in the left lead limb. HH (cm) was measured from the center of the two waist angles to the ground. SC (cm) was measured with a special measuring ruler as the circumference of the most prominent part of the middle part of the scrotum.

### 2.2. Data Preprocessing for Genetic Analysis

The screening criteria of qualified semen were as follows: SVPE ≥ 2 mL, SCPE ≥ 2 × 10^8^/mL, and SMPE ≥ 0.4. Three levels were involved in the season effects, where June, July, and August were the first level, January, February, and December were the second level, and the rest of the months were grouped into the third level. According to pedigree information obtained from the Dairy Data Center of China (https://www.holstein.org.cn) and the Canadian Dairy Network (https://www.cdn.ca), a total of 453 bulls were traced back to three generations, with 2,030 individuals included in the pedigree.

### 2.3. Two-Trait Genetic Analysis

A bivariate repeatability animal model was used to estimate variance and covariance components of semen and conformation traits using average information-restricted maximum likelihood (AI-REML) in the DMU package [13]. The model following the previous study [14, 15] is described as follows:

(1) $$\begin{array}{c} \left[\begin{array}{c} y_{1}\\ y_{2} \end{array}\right]=\left[\begin{array}{cc} X_{1} & 0\\ 0 & X_{2} \end{array}\right]\left[\begin{array}{c} b_{1}\\ b_{2} \end{array}\right]+\left[\begin{array}{cc} Z_{1} & 0\\ 0 & Z_{2} \end{array}\right]\left[\begin{array}{c} a_{1}\\ a_{2} \end{array}\right]+\left[\begin{array}{cc} W_{1} & 0\\ 0 & W_{2} \end{array}\right]\left[\begin{array}{c} p_{1}\\ p_{2} \end{array}\right]+\left[\begin{array}{c} e_{1}\\ e_{2} \end{array}\right], \end{array}$$

where *y*_1_ and *y*_2_ are the vectors of phenotypes (i.e., semen or conformation traits), *X*_1_ and *X*_2_ are the design matrices for fixed effects, *b*_1_ and *b*_2_ are the vectors of fixed effects (i.e., year-season and age effects), *Z*_1_ and *Z*_2_ are the design matrices for additive genetic effects, *a*_1_ and *a*_2_ are the vectors of additive genetic effects that satisfy *a*_1_ ~ *N*(0, *Aσ*_*a*_1__^2^) and *a*_2_ ~ *N*(0, *Aσ*_*a*_2__^2^), where *σ*_*a*_1__^2^ and *σ*_*a*_2__^2^ are additive genetic variances and *A* is the relationship matrix calculated using pedigree information, *W*_1_ and *W*_2_ are the design matrices for permanent environmental effects, *p*_1_ and *p*_2_ are vectors of permanent environmental effects that satisfy *p*_1_ ~ *N*(0, *Iσ*_*p*_1__^2^) and *p*_2_ ~ *N*(0, *Iσ*_*p*_2__^2^), where *σ*_*p*_1__^2^ and *σ*_*p*_2__^2^ are permanent environmental variances, and *e*_1_ and *e*_2_ are the vectors of random environmental effects that satisfy *e*_1_ ~ *N*(0, *Iσ*_*e*_1__^2^) and *e*_2_ ~ *N*(0, *Iσ*_*e*_2__^2^), where *σ*_*e*_1__^2^ and *σ*_*e*_2__^2^ are residual variances and *I* is the identity matrix.

The variance and covariance structure of the additive genetic, permanent environmental, and random environmental effects for two traits (i.e., semen or conformation traits) are

(2) $$\begin{array}{c} \left[\begin{array}{ccc} \begin{array}{cc} A\sigma_{a_{1}}^{2} & A\sigma_{a_{12}}\\ A\sigma_{a_{12}} & A\sigma_{a_{2}}^{2} \end{array} & \begin{array}{cc} 0 & 0\\ 0 & 0 \end{array} & \begin{array}{cc} 0 & 0\\ 0 & 0 \end{array}\\ \begin{array}{cc} 0 & 0\\ 0 & 0 \end{array} & \begin{array}{cc} I\sigma_{p_{1}}^{2} & I\sigma_{p_{12}}\\ I\sigma_{p_{12}} & I\sigma_{p_{2}}^{2} \end{array} & \begin{array}{cc} 0 & 0\\ 0 & 0 \end{array}\\ \begin{array}{cc} 0 & 0\\ 0 & 0 \end{array} & \begin{array}{cc} 0 & 0\\ 0 & 0 \end{array} & \begin{array}{cc} I\sigma_{e_{1}}^{2} & 0\\ 0 & I\sigma_{e_{2}}^{2} \end{array} \end{array}\right], \end{array}$$

where heritability (*h*^2^) and repeatability (*r*^2^) of the two traits are estimated as *h*_1_^2^ = *σ*_*a*_1__^2^/*σ*_*a*_1__^2^ + *σ*_*p*_1__^2^ + *σ*_*e*_1__^2^, *h*_2_^2^ = *σ*_*a*_2__^2^/*σ*_*a*_2__^2^ + *σ*_*p*_2__^2^ + *σ*_*e*_2__^2^, *r*_1_^2^ = *σ*_*a*_1__^2^ + *σ*_*p*_1__^2^/*σ*_*a*_1__^2^ + *σ*_*p*_1__^2^ + *σ*_*e*_1__^2^, and *r*_2_^2^ = *σ*_*a*_2__^2^ + *σ*_*p*_2__^2^/*σ*_*a*_2__^2^ + *σ*_*p*_2__^2^ + *σ*_*e*_2__^2^. Genetic correlation *r*_a_ and phenotypic correlation *r*_p_ are estimated as *r*_a_ = *σ*_*a*_12__/*σ*_*a*_1__*σ*_*a*_2__ and $r_{p}=\sigma_{a_{12}}+\sigma_{p_{12}}/\sqrt{\left(\sigma_{a_{1}}^{2}+\sigma_{p_{1}}^{2}+\sigma_{e_{1}}^{2}\right)\times \left(\sigma_{a_{2}}^{2}+\sigma_{p_{2}}^{2}+\sigma_{e_{2}}^{2}\right)}$, where *σ*_*a*_12__ and *σ*_*p*_12__ are covariances of the additive genetic and permanent environmental effects, respectively. The covariances of random environmental effects were not considered between semen and conformation traits, but were considered among semen traits or conformation traits.

## 3. Results

### 3.1. Statistical Description and Heritability of Semen and Conformation Traits

The statistical description of seven semen traits and nine conformation traits are presented in Table 2. The heritability, repeatability, and variance component estimates are presented in Table 3. It showed that semen traits had low to medium heritabilities ranging from 0.06 to 0.37, while conformation traits had relatively high heritabilities ranging from 0.23 to 0.69. TNMS, PTM, and SCPE traits exhibited the lower heritabilities of 0.06, 0.09, and 0.12, respectively. In contrast, AC, WH, SC, and HH traits exhibited higher heritabilities of 0.54, 0.59, 0.64, and 0.69, respectively. The repeatability ranges from 0.24 to 0.49 among semen traits and 0.27 to 0.76 among conformation traits (Table 3).

### 3.2. Genetic and Phenotypic Correlations of Semen and Conformation Traits

The genetic correlations (upper diagonal) and phenotypic correlations (lower diagonal) of semen and conformation traits are presented in Table 4. The phenotypic correlations among semen traits range from −0.20 to 0.72, with the highest observed correlation of SCPE with TNS and TNMS traits (0.72). For conformation traits, the phenotypic correlations range from 0.20 to 0.83, all showing positive correlations. The correlations of SM and PTM traits with conformation traits are generally weak or close to zero, while SVPE, SCPE, TNS, and TNMS traits exhibit positive correlations ranging from 0.07 to 0.48 (Table 4).

Genetic correlations among semen traits ranged from −0.34 to 0.80, where SVPE with TNS (0.79) and SCPE with TNMS (0.80) showed high correlations. Genetic correlations among conformation traits were all positive ranging from 0.18 to 0.88, where BW with CG (0.88), BW with AC (0.84), BW with BL (0.82), and BW with TC (0.82) showed high correlations. General high genetic correlations between semen traits and conformation traits were found largely from −1.00 to 1.00, where TNS with SC showed the highest correlation (1.00). Notably, genetic correlations for SC with SVPE, SCPE, TNS, and TNMS traits were 0.97, 0.98, 1.00, and 0.99, respectively, which indicates that the larger scrotal circumference can lead to a greater volume of semen, higher semen concentration, total number of sperm, and total number of motile sperm per ejaculation. Such high genetic correlations were also observed in WH and HH traits. Furthermore, BW also had a moderate genetic correlation with SVPE (0.94), TNS (0.93), and TNMS (0.89) traits (Table 4).

## 4. Discussion

Based on 56,980 semen records and 2,635 conformation records of 453 Chinese Holstein bulls over the last 10 years, the estimated genetic parameters of our study were favorable and comparable. For semen traits, the SVPE (0.36), SMPE (0.27), SCPE (0.12), PAS (0.37), and TNMS (0.06) heritabilities estimated in our study (Table 3) were similar to those of other studies (0.04∼0.43) [3, 8, 9, 16–19]. Carvalho Filho et al. [20] reported a quite low heritability of SMPE (only 0.05) suggesting the significant influence of it by the unstable environmental factors and the high phenotypic variability. However, the PAS (0.37) and TNS (0.32) heritabilities (Table 3) were quite higher than those of the beef cattle (0.03 and 0.13) as reported by Butler et al. [19] and Berry et al. [3]. The higher estimates of heritability in our study than in other studies were likely influenced by the limited repeated phenotypic records that made it hard to estimate the larger variance component for the permanent environmental effect than for the additive genetic effect [21–24].

For conformation traits, the HH heritability (0.69) was consistent with the results of Arango et al. [25]; however, the BW heritability (0.33) was clearly lower than the adult weight (0.75) of Hereford cattle [26]. Obviously, different data sources, sample sizes, and applied models may result in different estimated heritabilities of the same conformation traits [27]. Several previous studies also indicated that SC heritability from 0.40 to 0.81 [20, 28, 29] was consistent with our study (0.64) (Table 3). The high estimated heritabilities of conformation traits indicate that genetic improvement of semen production and quality by selection could be feasible if their genetic correlations with conformation traits are high enough.

The findings of genetic correlations among conformation traits underscore a robust relationship in the growth patterns across various body dimensions, showing the notable correlations of BW with BL at 0.71, CG at 0.83, and AC at 0.79 (Table 4). It highlights the significant interdependence in the development of distinct body parts and emphasizes the impact of specific dimensions on the overall conformation of each individual [27]. Moreover, our study reveals the strong genetic correlations ranging from 0.79 to 0.97 between semen and conformation traits. It indicates the significant genetic interconnection between seminal production and quality and various aspects of conformation in Chinese Holstein bulls.

Many studies have also reported the positive correlations between these two types of traits, such as 0.36 for SC with sperm motility in Angus cattle [30] and 0.20 and 0.77 for SC with semen volume and contraction, respectively, in Hereford cattle [9]. Berry et al. [3] also showed the high genetic correlations of SC with ejaculation volume, concentration, and motility (0.20, 0.77, and 0.76) in dairy and beef cattle. It was worth mentioning the quite high genetic correlations for SC with SVPE (0.97), SCPE (0.98), TNS (1.00), and TNMS (0.99) traits (Table 4) in the current study. The elevated genetic correlations suggest that the selection for favorable conformation traits could positively contribute to the enhancement of seminal production and quality, particularly for the SVPE trait. Therefore, the selection of those conformation traits could help improve semen production and quality in Holstein bulls.

Some other conformation traits were related to semen traits in strong positive correlation, specifically for WH with SVPE (0.88), SCPE (0.87), TNS (0.93), and TNMS (0.91) traits and HH with SVPE (0.87), SCPE (0.85), TNS (0.92), and TNMS (0.90) traits. It indicates that superior morphological conformation may underlie much of the variation in the semen volume, concentration, and number properties. Similarly, we identified the robust genetic correlation between BW and TNS and TNMS at 0.93 and 0.89, respectively (Table 4). These results highlight the close linkage between specific body dimensions and reproductive characteristics. Understanding these relationships is pivotal for comprehensive breeding strategies and substantial insights into the particular selection for conformation traits positively influencing both semen production and quality in Chinese Holstein bulls. Undoubtedly, the potential unstable and unfavorable estimates still exist, so further study for more accurate estimations is needed by using an enlarged population size when more phenotypic records and pedigree information are enrolled.

## 5. Conclusion

In summary, our study provides the estimated heritabilities and genetic correlations of semen and conformation traits in Chinese Holstein bulls. Of note, positive genetic correlations of conformation traits were found with SVPE, SCPE, TNS, and TNMS, especially for SC, BW, WH, and HH traits (0.87∼1.00). These results indicate a robust relationship of the growth traits with semen traits, so that the genetic improvement of semen production and quality traits by selection could be feasible. Especially, SC could be used as an indicator trait for indirect selection, or as an assistant trait to improve selection accuracy for semen production and quality. Such insights shed light on potential synergies between reproductive and conformation traits that emphasize the importance of considering both parts in genetic improvement programs. However, the enlarged population size is still needed to guarantee more accurate estimations in the further practical genetic improvement programs in Holstein bulls.

## Figures and Tables

**Table 1:** Quality control procedures of semen traits and conformation traits.

Step	Content	Criterion	Sample size	
			Semen trait	Conformation trait
1	Semen and conformation traits	Raw data	66,260	3,642
2	SVPE	≥2 mL	65,600	3,642
3	SCPE	≥2 × 10^8^/mL	65,412	3,642
4	SMPE	≥0.4	64,087	3,642
5	Semen traits	Removal of unmeasured data	64,072	3,642
6	Conformation traits	Removal of data before 2014	64,072	2,881
7	Semen and conformation traits	Removal of data without birth date	56,980	2,774
8	Conformation traits	Removal of data without completion of three-generation pedigree	56,980	2,635

SVPE = semen volume per ejaculation; SCPE = semen concentration per ejaculation; SMPE = sperm motility per ejaculation.

**Table 2:** Statistical description of semen and conformation traits.

Semen and conformation trait	Sample size for each trait	Mean ± standard deviation	Coefficient of variation (%)	Maximum	Minimum
SVPE (mL)	56980	6.52 ± 2.25	34.56	21.42	2.00
SMPE (%)	56975	62.91 ± 7.47	11.87	85.00	40.00
SCPE (10^8^/mL)	56975	13.25 ± 4.55	34.32	36.73	2.00
PAS (%)	6094	12.86 ± 3.89	30.29	38.00	0.00
TNS (10^8^)	56975	87.01 ± 42.7	49.08	392.32	4.68
TNMS (10^8^)	56975	55.23 ± 28.21	51.07	270.81	2.14
PTM (%)	25594	37.01 ± 7.67	20.71	62.00	1.00
BW (kg)	2550	616.66 ± 189.84	30.79	1287.00	330.00
WH (cm)	2614	149.66 ± 10.81	7.22	190.00	127.00
BL (cm)	2603	183.27 ± 22.36	12.20	252.00	133.00
CG (cm)	2607	201.42 ± 21.21	10.53	284.00	155.00
AC (cm)	2607	234.19 ± 20.98	8.96	318.00	186.00
TC (cm)	2553	21.42 ± 1.77	8.28	28.00	17.00
PW (cm)	2438	13.35 ± 1.61	12.04	23.00	11.00
HH (cm)	2609	151.49 ± 9.22	6.09	185.00	120.00
SC (cm)	2572	37.52 ± 3.49	9.30	51.50	26.00

SVPE = semen volume per ejaculation; SMPE = sperm motility per ejaculation; SCPE = semen concentration per ejaculation; PAS = percentage of abnormal sperms; TNS = total number of spermss; TNMS = total number of motile sperms; PTM = postthaw motility; BW = body weight; WH = withers height; BL = body length; CG = chest girth; AC = abdominal circumference; TC = tube circumference; PW = pin width; HH = hip height; SC = scrotal circumference.

**Table 3:** Heritabilities (*h*^2^), repeatability (*r*^2^), and variance component estimates of direct additive genetic (*σ*_*a*_^2^), permanent environment (*σ*_*p*_^2^), and environmental (*σ*_*e*_^2^) variances using the bivariate repeatability animal model.

Semen and conformation trait	*σ*_*a*_^2^	*σ*_*p*_^2^	*σ*_*e*_^2^	*h* ^2^	Maximum *h*^2^	Minimum *h*^2^	*r* ^2^	Maximum *r*^2^	Maximum *r*^2^
SVPE (mL)	1.76	0.61	2.47	0.36	0.43	0.32	0.49	0.51	0.45
SMPE (%)	5.75	3.88	12.09	0.27	0.42	0.20	0.44	0.45	0.42
SCPE (10^8^/mL)	7.44	12.39	41.21	0.12	0.22	0.10	0.32	0.33	0.32
PAS (%)	655.87	119.15	1004.24	0.37	0.40	0.33	0.43	0.46	0.40
TNS (10^8^)	243.79	65.95	451.53	0.32	0.38	0.28	0.41	0.43	0.38
TNMS (10^8^)	0.67	2.19	9.01	0.06	0.23	0.01	0.24	0.26	0.23
PTM (%)	5.68	11.34	46.80	0.09	0.22	0.02	0.27	0.33	0.26
BW (kg)	2265.78	62.55	4487.05	0.33	0.44	0.26	0.34	0.48	0.28
WH (cm)	30.60	1.57	17.86	0.59	0.77	0.48	0.62	0.79	0.51
BL (cm)	29.21	1.74	82.96	0.25	0.34	0.17	0.27	0.34	0.21
CG (cm)	35.98	1.06	70.89	0.33	0.43	0.25	0.34	0.46	0.28
AC (cm)	86.79	2.75	69.72	0.54	0.64	0.49	0.56	0.69	0.49
TC (cm)	0.34	0.14	0.71	0.28	0.42	0.12	0.40	0.50	0.33
PW (cm)	0.36	0.07	1.13	0.23	0.30	0.10	0.27	0.34	0.23
HH (cm)	35.94	1.19	13.68	0.69	0.81	0.63	0.71	0.83	0.64
SC (cm)	6.97	1.28	2.44	0.64	0.75	0.50	0.76	0.84	0.71

SVPE = semen volume per ejaculation; SMPE = sperm motility per ejaculation; SCPE = semen concentration per ejaculation; PAS = percentage of abnormal sperms; TNS = total number of sperms; TNMS = total number of motile sperms; PTM = postthaw motility; BW = body weight; WH = withers height; BL = body length; CG = chest girth; AC = abdominal circumference; TC = tube circumference; PW = pin width; HH = hip height; SC = scrotal circumference.

**Table 4:** Genetic correlations (upper diagonal) and phenotypic correlations (lower diagonal) of semen and conformation traits.

Trait	SVPE	SMPE	SCPE	PAS	TNS	TNMS	PTM	BW	WH	BL	CG	AC	TC	PW	HH	SC
SVPE		—	—	—	0.79	0.76	—	0.94	0.88	0.92	0.85	0.89	0.86	0.79	0.87	0.97
SMPE	—		—	—	—	—	0.70	−0.52	−0.49	−0.16	−0.48	−0.53	−0.55	−0.49	−0.58	−0.55
SCPE	—	—		−0.25	0.76	0.80	−0.09	0.75	0.87	0.92	0.69	0.72	0.50	0.75	0.85	0.98
PAS	—	—	−0.05		—	—	−0.34	−0.83	−0.81	−0.99	−0.77	−0.69	−1.00	−0.54	−0.72	−0.60
TNS	0.69	—	0.72	—		—	0.02	0.93	0.93	—	0.89	0.89	0.83	0.83	0.92	1.00
TNMS	0.64	—	0.72	—	—		0.15	0.89	0.91	—	0.83	—	0.79	0.79	0.90	0.99
PTM	—	0.35	−0.16	−0.14	−0.20	−0.15		−0.60	−0.35	−0.41	−0.49	−0.68	0.10	−0.35	−0.56	−0.50
BW	0.27	−0.03	0.16	−0.08	0.32	0.26	−0.02		0.58	0.82	0.88	0.84	0.82	0.40	—	0.56
WH	0.40	−0.03	0.23	−0.10	0.44	0.39	−0.01	0.67		0.78	0.72	0.47	—	0.42	—	—
BL	0.24	0.00	0.18	−0.06	—	—	0.00	0.71	0.67		0.72	0.59	—	0.21	0.81	—
CG	0.26	−0.01	0.15	−0.06	0.30	0.25	−0.01	0.83	0.71	0.63		0.80	—	0.31	—	—
AC	0.32	−0.06	0.15	−0.08	0.34	—	−0.05	0.79	0.55	0.55	0.75		—	0.39	0.61	0.46
TC	0.26	−0.03	0.10	−0.08	0.28	0.22	0.02	0.67	—	—	—	—		0.18	—	—
PW	0.21	−0.03	0.07	−0.01	0.21	0.17	−0.01	0.39	0.38	0.31	0.37	0.34	0.31		0.47	0.24
HH	0.41	−0.06	0.22	−0.10	0.44	0.37	−0.04	—	—	0.65	—	0.58	—	0.39		—
SC	0.35	−0.04	0.35	−0.02	0.48	0.42	−0.07	0.48	—	—	—	0.45	—	0.20	—	

SVPE = semen volume per ejaculation; SMPE = sperm motility per ejaculation; SCPE = semen concentration per ejaculation; PAS = percentage of abnormal sperms; TNS = total number of sperms; TNMS = total number of motile sperms; PTM = postthaw motility; BW = body weight; WH = withers height; BL = body length; CG = chest girth; AC = abdominal circumference; TC = tube circumference; PW = pin width; HH = hip height; SC = scrotal circumference; “—” = no result.

## Data Availability

All data from the current study are consolidated in tables and Supplementary Materials presented in this article. If further information is required, please contact the corresponding author: Jianbin Li, msdljb@163.com.

## Abbreviations

AC: Abdominal circumference
AI-REML: Average information-restricted maximum likelihood
BL: Body length
BW: Body weight
CG: Chest girth
HH: Hip height
PAS: Percentage of abnormal sperm
PTM: Postthaw motility
PW: Pin width
SC: Scrotal circumference
SCPE: Semen concentration per ejaculation
SD: Standard deviation
SE: Standard error
SMPE: Sperm motility per ejaculation
SVPE: Semen volume per ejaculation
TC: Tube circumference
TNMS: Total number of motile sperms
TNS: Total number of sperms
WH: Withers height.

## Supplementary Materials

Supplementary Materials Supplementary File 1: the detailed information of seven semen traits and nine conformation traits.
